# Novel and Specific MRI Features Indicate the Clinical Features of Patients With Rare Hepatic Tumor Epithelioid Hemangioendothelioma

**DOI:** 10.3389/fonc.2022.729177

**Published:** 2022-01-27

**Authors:** Wei Zhang, Hongtao Zhang, Yanwei Zhong, Keming Zhang, Huifang Kong, Linxiang Yu, Yan Chen, Yili Bai, Zhenyu Zhu, Yongping Yang, Xudong Gao

**Affiliations:** ^1^ Department of Hepatology and Department of Radiology, The 5th Medical Center of PLA General Hospital, Beijing, China; ^2^ Department of Hepatobiliary Surgery, Peking University International Hospital, Beijing, China; ^3^ Beijing Shijitan Hospital, Capital Medical University, Beijing, China

**Keywords:** hepatic epithelioid hemangioendothelioma (HEHE), dynamic contrast-enhanced magnetic resonance imaging (MRI), liver tumor, hepatic vein branches, extrahepatic metastasis

## Abstract

**Objective:**

To investigate the MRI features and clinical significance of hepatic epithelioid hemangioendothelioma (HEHE).

**Methods:**

Clinical records and MRI findings were retrospectively evaluated in nine HEHE patients from May 2010 to January 2020.

**Result:**

There were 121 lesions in nine patients with a predominantly peripheral distribution. Five lesions (4.13%) in two patients (22.22%) had evidence of capsular retraction, and three patients had lung metastasis (33.33%). Dynamic contrast-enhanced MRI showed progressive enhancement, mainly in two ways: ring enhancement with hypovascularity in four patients (44.44%) and ring enhancement with hypervascularity in five patients (55.56%). Imaging demonstrated a multilayer ring appearance, which was typically observed on T2-weighted imaging (T2WI). The most common appearance consisted of two layers of varying signal, with some images displaying up to four layers. There were significant differences in the size of lesions between different layers of multilayer ring appearance (*p* < 0.001). All lesions exhibited a two-layer appearance on diffusion-weighted imaging (DWI), with hyperintensity at the periphery and a slightly high signal at the center (except for those with a single layer on T2WI). The “vascular penetration sign” was observed in most lesions, and the blood vessels of 112 lesions (92.56%) were portal vein branches, and five (4.13%) were hepatic vein branches. Pulmonary metastasis was found in three patients with the “vascular penetration sign” of hepatic vein branches.

**Conclusion:**

The multilayer ring appearance on T2WI, the “vascular penetration sign”, and the two enhancement patterns may be of great significance in the diagnosis and treatment of HEHE. The “vascular penetration sign” of hepatic vein branches may indicate extrahepatic metastasis.

## Introduction

Hepatic epithelioid hemangioendothelioma (HEHE) is a rare vascular malignancy of the liver that carries a low malignant potential (incidence rate of 1/1 million population) (1–3). Originating from mesenchymal tissue, the malignant potential of HEHE is between benign hemangioma and malignant angiosarcoma (4). Because of its uncertainty, HEHE is commonly misdiagnosed as primary or secondary liver cancer. The tumor generally affects patients of all ages, and the highest incidence is between 35 and 45 years old (5–7), with a male-to-female ratio of 2:3 (8, 9). Etiologic factors are currently unknown, and although several risk factors have been proposed, none has been confirmed to increase the risk of developing HEHE, including the hepatitis virus and chronic liver diseases (2, 10).

After early diagnosis, effective treatment options include surgical resection and liver transplantation. However, most patients are diagnosed with HEHE in the final stage when surgical resection is not possible (9, 11, 12). Several studies have advocated that patients with multiple nodules can undergo liver transplantation, and some patients with metastasis may still benefit from liver transplantation; thus, metastasis is not an absolute contraindication to liver transplantation (5, 9, 12). Other treatment options such as radiotherapy and chemotherapy, which are not effective for most other liver tumors (13, 14), do not show favorable prognoses (9, 15).

Imaging examination is one of the main methods for diagnosing liver lesions (16, 17). However, the diagnosis of HEHE can be difficult (18, 19), and pathological examination is often needed. Early detection of HEHE based on imaging findings is one of the main clinical challenges in the study of this disease (19).

Numerous studies have explored the imaging features of HEHE and demonstrated several characteristics, such as early peripheral ring enhancement, capsule retraction, and the “lollipop” sign (19–23). However, further studies are required to improve diagnosis and treatment. We retrospectively reviewed nine patients with 121 lesions who were admitted to the Fifth Medical Center of PLA General Hospital in the past 10 years. The purpose of this study was to review and analyze imaging findings from the liver MRI of HEHE patients to enable more accurate diagnoses and demonstrate clinical significance.

## Materials and Methods

### General Information

Clinical records and MRI findings of all initially diagnosed HEHE patients admitted to the Fifth Medical Center of PLA General Hospital from May 2010 to January 2020 were reviewed. Nine patients (**Table 1**) were admitted, of whom none received invasive or antitumor treatments, such as radiotherapy and chemotherapy. After admission, all patients underwent abdominal MRI and lung CT. Liver biopsies were performed, and all diagnoses were confirmed as HEHE.

**Table 1:** Summary of general information and clinical features of patients.

Patient	Sex	Age (years)	History of liver disease	Presenting symptom(s)	Tumor markers*	CD31	CD34	Factor VIII
1	Male	43	None	Upper right quadrant pain	Negative	+	++	+
2	Male	32	None	Asymptomatic	Negative	+	++	+
3	Female	45	None	Cough	Negative	+	++	+
4	Female	35	None	Abdominal pain	Negative	+	+	+
5	Male	49	Alcoholic liver disease	Abdominal distension	Negative	+	+	+
6	Male	59	Hepatitis B	Asymptomatic	Negative	+	++	+
7	Male	35	None	Abdominal pain	Negative	+	++	+
8	Female	43	None	Abdominal pain	Negative	+	++	+
9	Male	50	None	Abdominal pain	Negative	+	+	+

+/++, positive/strong positive.

*Tumor markers including AFP, CA125, CA199, CA724, and CEA.

### Abdominal MRI

MRI examinations were performed on a 3.0-T system (General Electric Company (GE) HDXt). For T2WI, respiratory-triggered fat-suppressed fast spin-echo (FSE) was used with the following parameters: repetition time (TR)/echo time (TE), 9,000/81.9 ms; matrix, 256 × 256–320 × 256; 6–10-mm thickness with a layer spacing of 1–2 mm; field of view (FOV), 34 × 40–40 × 40 cm. Then, transverse T1-weighted in-phase and out-of-phase sequences were performed using the following parameters: TR/TE, 3.8/(2.2;1.1) ms; matrix, 256 × 256–320 × 256; 5-mm thickness with an interpolated section thickness of 2.5 mm; FOV, 34 × 40–40 × 40 cm. Transverse breath-hold diffusion-weighted imaging (DWI) was obtained using b-values of 0 and 800 s/mm^2^ with the following parameters: TR/TE, 4615/60 ms; matrix, 160 × 128; 6–10-mm thickness with a layer spacing of 1–2 mm; FOV, 34 × 40–40 × 40 cm. Three-dimensional fat-saturated T1-weighted dynamic contrast enhancement sequences were also performed (arterial phase: early and late phases, 18–22 s, portal venous phase, 60 s, coronal balance phase, 3–5 min, and delayed phase, 5 min). The injection rate of the high-pressure injector was 1.5–2.5 ml/s at a dose of 0.1 mmol/kg. The parameters were as follows: TR/TE, 3.9/1.7 ms; matrix, 256 × 256–320 × 256; 5-mm thickness with an interpolated section thickness of 2.5 mm; FOV, 34 × 40–40 × 40 cm.

### Lung CT

The LightSpeed volume CT (VCT) spiral CT scanner by GE (United States) was used. Patients were scanned in a supine position with the head advanced and the breath held at the end of inspiration. CT scanning parameters were as follows: tube voltage, 100–120 kV; tube current, 150 mA; pitch, 0.98 mm; matrix, 512 × 512; slice thickness, 5.0 mm; FOV, 350 × 350 mm; multiplanar reconstruction with a thickness of 1.25 mm. For the lung standard window, the window width was 1,500 HU, and the window level was −500 HU. For the mediastinal window, the window width was 350 HU, and the window level was 50 HU.

### Image Analysis

All images were independently reviewed by two radiologists who had 10 and 8 years of experience in the interpretation of abdominal MR images. Final decisions were reached by consensus. The “vascular penetration sign” was defined as the penetration of the terminal branch of the portal or hepatic vein from the edge to the interior of the tumor that extends a certain distance on dynamic contrast enhancement.

### Pathological Examination

All patients underwent liver biopsy, which included six percutaneous ultrasound-guided biopsies and three CT-guided biopsies. The histopathological evaluation included routine H&E and immunohistochemical staining. Endothelial markers included CD31, CD34, and a factor VIII-related antigen. Pathological examinations and specimen handling were performed by experienced liver pathologists.

### Statistical Analysis

SPSS 13.0 software was used for data analyses. Continuous variables are expressed as means ± SDs. Comparisons between groups were performed using one-way ANOVAs. A *p* < 0.05 was considered statistically significant.

## Results

### Patients

There were a total of nine patients (**Table 1**), which included six males (6/9, 66.67%) and three females (3/9, 33.33%) aged 43.4 ± 8.60 years. One patient had a history of hepatitis B, and one had a history of alcoholic liver disease. The most common presenting symptoms were upper abdominal discomfort and right upper quadrant abdominal pain. One patient’s primary symptom was a cough, which may have been related to lung metastasis. Two patients were asymptomatic, whose lesions were found by physical examination. Tumor biomarkers, including AFP, CA125, CA199, CA724, and CEA, were normal in all patients. Immunohistochemical staining showed that patients with pathologically proven HEHE were CD31- and CD34-positive.

All nine patients underwent abdominal MRI and lung CT. Eight patients had multiple lesions (8/9, 88.89%), and one patient had a single lesion (1/9, 11.11%). There were a total of 121 nodules with a predominantly peripheral distribution (**Table 2**). The shape of most nodules was round. Nine lesions (9/121, 7.44%) in four patients (4/9, 44.44%) showed nodules that had coalesced to form confluent lesions during development (six lesions: each lesion was formed by the fusion of two nodules; 3 lesions: each lesion was formed by fusion of 3 or more nodules); five lesions (5/121, 4.13%) in two patients (2/9, 22.22%) had evidence of capsular retraction; three patients had pulmonary metastasis (3/9, 33.33%).

**Table 2:** Summary of MRI findings in nine patients.

Patient	Number of lesions	Distribution	Shape	Liver capsular	Coalescence	Vascular penetration sign	Extrahepatic metastasis	Number of layers in multilayer ring appearance	Enhancement pattern
1	30	Predominantly peripheral	Round-like	No retraction	3	Portal veins (27 lesions) No blood vessel (3 lesions)	None	Two-layer (25 lesions); single layer (5 lesions)	Hypovascularity and progressive enhancement
2	5	Predominantly peripheral	Round-like	No retraction	0	Portal veins (2 lesions) Hepatic veins (3 lesions)	Pulmonary	Two-layer (5 lesions)	Hypovascularity and progressive enhancement
3	4	Predominantly peripheral	Round-like	No retraction	0	Portal veins (4 lesions) Hepatic veins (1 lesion)*	Pulmonary	Two-layer (4 lesions)	Hypovascularity and progressive enhancement
4	5	Predominantly peripheral	Round-like	No retraction	1	Portal veins (4 lesions) Hepatic veins (1 lesion)	Pulmonary, retroperitoneal lymph node	Four-layer (4 lesions); two-layer (1 lesion)	Hypervascularity and progressive enhancement
5	18	Predominantly peripheral	Round-like	No retraction	2	Portal veins (18 lesions)	None	Three-layer (18 lesions)	Hypervascularity and progressive enhancement
6	1	Peripheral	Round-like	No retraction	0	Portal veins (1 lesion)	None	Two-layer (1 lesion)	Hypervascularity and progressive enhancement
7	21	Predominantly peripheral	Round-like	Retraction	0	Portal veins (19 lesions) No blood vessel (2 lesions)	None	Two-layer (21 lesions)	Hypervascularity and progressive enhancement
8	32	Predominantly peripheral	Round-like	No retraction	3	Portal veins (32 lesions)	None	Two-layer (18 lesions); three-layer (14 lesions)	Hypervascularity and progressive enhancement
9	5	Peripheral	Round-like	Retraction	0	Portal veins (5 lesions)	None	Two-layer (5 lesions)	Hypovascularity and progressive enhancement

*Vascular penetration sign of 1 lesion included portal and hepatic veins.

### Multilayer Ring Appearance

**Figure 1:**
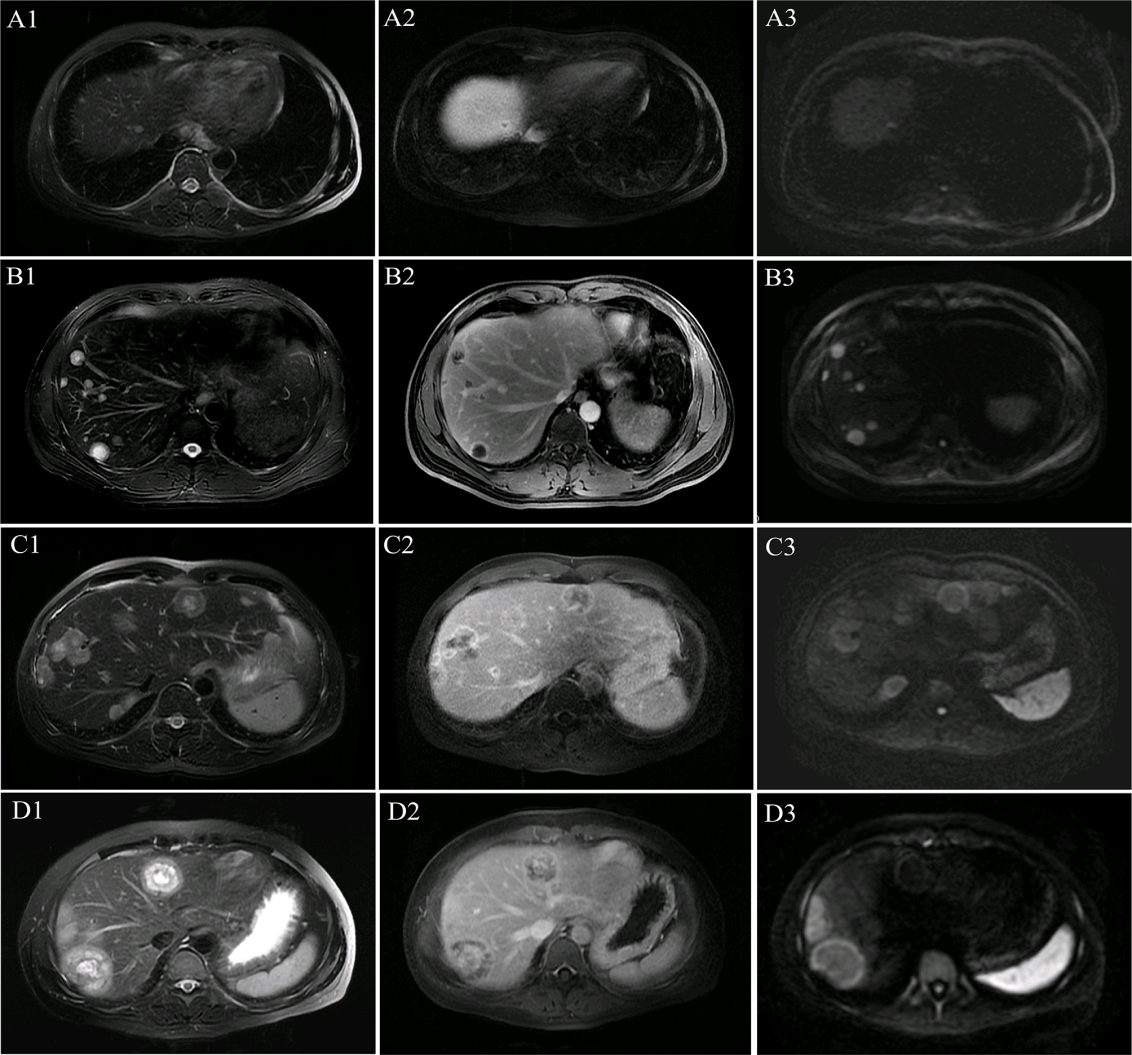
Multilayer ring appearance. The multilayer ring appearance was seen most clearly in T2-weighted imaging (T2WI). The multilayer ring appearance with alternating high and low signal intensity (except for in the single-layer structure). **(A1–A3)** MR images of lesions with a single layer in patient 1. **(B1–B3)** MR images of lesions with two layers in patient 1. **(C1–C3)** MR images of lesions with three layers in patient 5. **(D1–D3)** MR images of lesions with four layers in patient 4. **(A1)** T2WI shows a single layer with slightly high signal intensity. **(A2)** Single-layer ring appearance on the delayed phase with peripheral enhancement and low central signal. **(A3)** Single-layer ring appearance on the diffusion-weighted imaging (DWI) image shows slightly high signal intensity. **(B1)** T2WI shows a two-layer ring appearance with slightly high peripheral and high central signal intensity. **(B2)** Two-layer ring appearance on the delayed phase with peripheral enhancement and low central signal. **(B3)** Two-layer ring appearance on the DWI image shows high peripheral signal intensity and slightly high central signal intensity. **(C1)** T2WI shows a three-layer ring appearance with slightly high peripheral signal intensity (the first layer), high central signal intensity (the third layer), and intermediate (the second layer) signal intensity, which demonstrates lower signal intensity comparing with that of the other two layers. **(C2)** Three-layer ring appearance on the delayed phase with obvious peripheral enhancement (the first layer), low central signal intensity (the third layer), and intermediate (the second layer) slight enhancement, which was not as clear as on T2WI. **(C3)** Three-layer ring appearance on the DWI image shows peripheral hyperintensity (the first layer) and slightly high signal intensity for the other layers. **(D1)** T2WI shows a four-layer ring appearance with slightly high peripheral signal intensity (the first layer), high signal intensity in the adjacent second layer, and slightly high signal intensity in the third layer and high central signal intensity (the fourth layer). **(D2)** Four-layer ring appearance on the delayed phase with obvious peripheral enhancement (the first layer), hypo-enhancement in the second adjacent layer, and hyper-enhancement in the third layer with slight central hypo-enhancement, which was not as clear as those on T2WI. **(D3)** Four-layer ring appearance on the DWI image shows peripheral hyperintensity (the first layer) and slightly high signal intensity for the other layers.

All patients showed a multilayer circular structure on MRI, which we called a “multilayer ring appearance”. This feature was seen most clearly in T2WI (**Figure 1**) and was also found in several lesions in the delayed phase but not as clear and uniform as in T2WI (**Figure 1**). To better understand this feature, we analyzed the T2WI data.

A multilayer ring appearance consisting of a core was more commonly seen in the two-layer structure but could be observed in up to four layers. The layer structure of nodules varied across patients. For example, the MRI of patient 8 (32 lesions) showed several lesions with a two-layer ring appearance (18 lesions) and other lesions with three layers (14 lesions). The multilayered ring appearance showed an alternation between high and low signal intensity (except for lesions with a single layer; **Figure 1**). There were five lesions (patient 1) with a single-layer structure with a median size (range) of 0.7 (0.4–1.8) cm; 80 lesions (in eight patients; all patients except patient 5) with a two-layer structure and a median size (range) of 1.5 (0.5–8.1) cm; 32 lesions (in two patients: patients 5 and 8) with a three-layer structure and a median size (range) of 3.1 (1.6–3.5) cm; and four lesions (in 1 patient: patient 4) with a four-layer structure with a median size (range) of 5.0 (4.2–5.3) cm.

**Figure 2:**
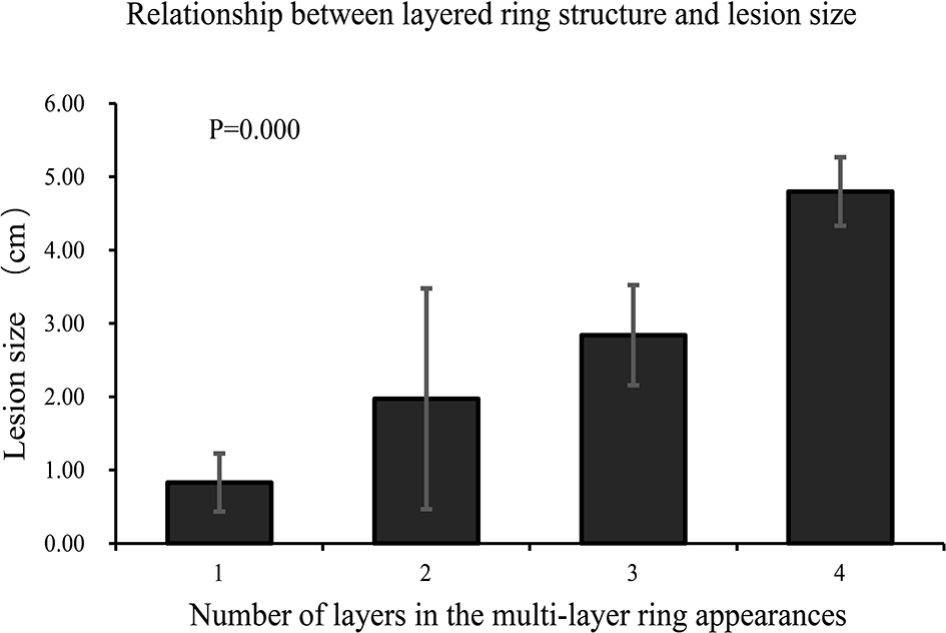
Comparison of lesion size between different multilayer ring appearances. The lesions were grouped according to the number of layers of the multilayer ring appearance. There were significant differences between groups (*p* < 0.001). The number of layers increased as the size of the lesion increased.

Comparisons of lesion size between different multilayer ring appearances (**Figure 2**) showed significant differences between every pair of different ring structure layers, *p*-values <0.001. As the number of layers increased, the size of lesions increased, which suggested that the formation of the multilayer ring structure was related to the growth of tumors. However, regardless of the number of layers of the ring structure shown on T2WI (except for a single layer), the DWI showed a target appearance that consisted of a core with a slightly hyperintense signal and a peripheral halo with high signal intensity (**Figure 1**).

### Vascular Penetration Sign

**Figure 3:**
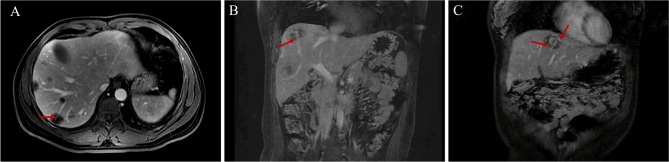
Vascular penetration sign. **(A, B)** The vascular penetration sign in transverse and coronal MR images. **(C)** Vascular penetration sign with the portal and hepatic veins in patient 3. The terminal branches of the portal vein are at the bottom, and the branches of the left hepatic vein are at the top.

Blood vessels penetrated most lesions and extended to a certain distance, which was clearer in the portal venous and delayed phases of the MRI (**Table 2** and **Figure 3**). We called this the “vascular penetration sign”. In this sign, all blood vessels were identified as veins, which were mainly small branches of the terminal of the portal vein. Of the 121 lesions studied, blood vessels in 112 lesions (112/121, 92.56%) were portal vein branches, and those in five lesions (5/121, 4.13%) were hepatic vein branches. In one lesion, there were two blood vessels, which included branches of the portal and hepatic veins. Five lesions show no blood vessel penetration, and the sizes were 0.4, 0.5, 0.4, 0.6, and 0.4 cm, which may be related to the size and the scanning section of the lesions.

The blood vessels of several lesions in three patients (patients 2, 3, and 4) were portal vein branches or hepatic veins. In patient 2, among the five lesions, the blood vessels of two lesions were portal vein branches, and those of three lesions were hepatic vein branches. In patient 3, among the four lesions, the blood vessels of three lesions were portal vein branches, and those of one lesion included both hepatic vein and portal vein branches. In patient 4, among the five lesions, the blood vessels of four lesions were portal vein branches, and those of one lesion were hepatic vein branches. The chest CTs of these three patients indicated lung metastasis. All of the “vascular penetration signs” in the other six patients involved portal vein branches rather than hepatic vein branches and were not lung metastases. Thus, the “vascular penetration sign” with hepatic vein branches may be related to lung metastasis.

### Two Major Enhancement Patterns

**Figure 4:**
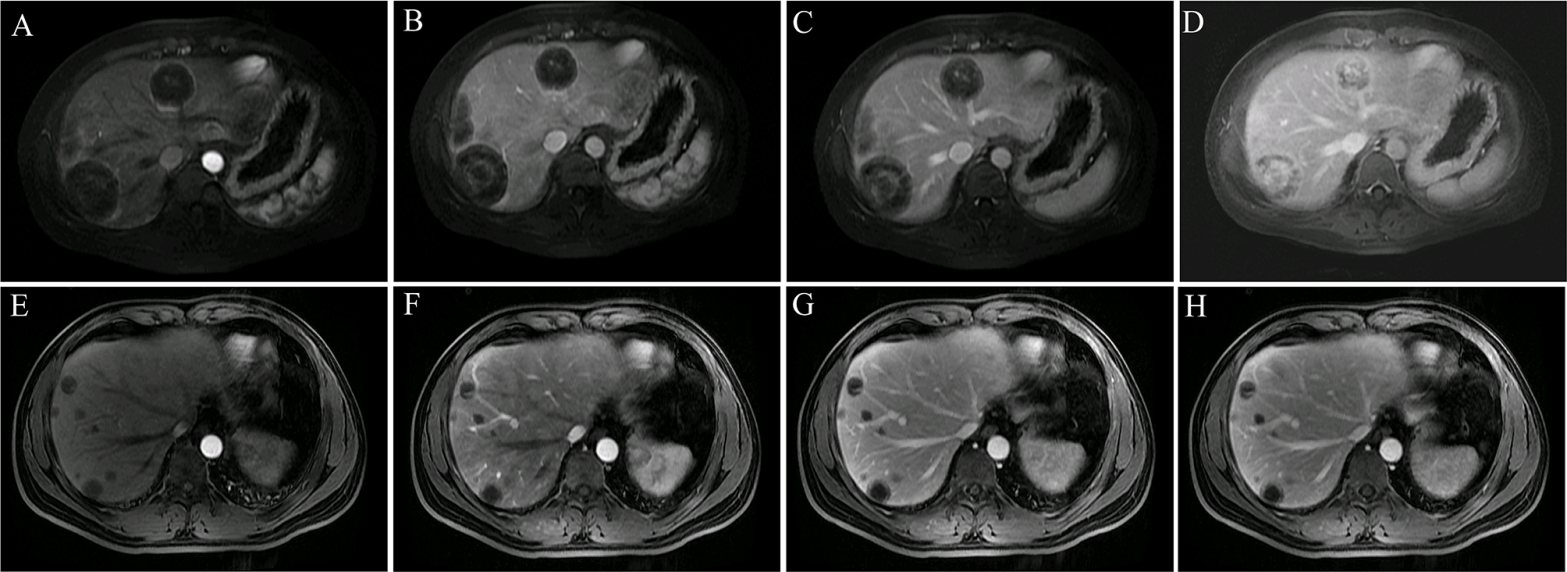
Two major enhancement patterns. **(A–D)** Tumors showed ring-like enhancement with hypervascularity and progressive enhancement (patient 4). The ring-like and local enhancements of the lesion in the early arterial **(A)** and late arterial **(B)** phases were higher than those of the liver parenchyma, and the lesion in the portal vein **(C)** and delayed phase **(D)** showed progressive enhancement and obvious layer-ring enhancement. **(E–H)** Tumors showed ring-like enhancement with hypovascularity and progressive enhancement (patient 1). The overall enhancement of the lesion in the early arterial **(E)**, late arterial **(F)**, portal **(G)**, and delayed phases **(H)** was significantly lower than that of the liver parenchyma. During the dynamic enhancement process, the lesion showed gradual ring enhancement in the periphery, and the central enhancement was not obvious.

Dynamic contrast-enhanced MRI (**Figure 4**) showed two major enhancement patterns in all patients: 1) in the early and late arterial, portal venous, and delayed phases, tumors showed ring enhancement with hypovascularity and progressive enhancement. The nodules appeared hypo-enhanced relative to normal liver parenchyma, and dynamic contrast-enhanced images showed gradual enhancement peripherally with no obvious hyper-enhancement core. This pattern was observed in four patients (4/9, 44.44%). 2) In the early and late arterial phases, tumors showed ring enhancement with hypervascularity and progressive enhancement. The nodules appeared locally hyper-enhanced relative to liver parenchyma, and in the portal venous and delayed phases, images showed gradual enhancement as a “multilayer ring appearance”. This pattern was observed in five patients (5/9, 55.56%). Thus, we observed two distinct patterns in this study.

## Discussion

HEHE is a rare vascular low-grade malignant tumor of the liver. It is frequently clinically misdiagnosed. We retrospectively studied nine patients with pathologically proven HEHE. Patients in this study were mainly male, which was different from previous reports (8, 9). The reason may be related to the small number of patients. Most patients had multiple lesions, and only one patient had a single lesion (11.11%). Combined with the clinic, a detailed analysis of MR images of every lesion was performed, and several findings provided diagnostic value and clinical significance.

Several studies have suggested that nodule coalescence and capsular retraction are characteristics of HEHE (18, 20, 24). However, in our study of nine patients and a total of 121 lesions, few lesions showed nodule coalescence, and capsular retraction was found in only five lesions (5/121, 4.13%) in two patients (2/9, 22.22%). Therefore, further research is needed to identify more significant features for HEHE diagnosis and treatment.

Dynamic contrast-enhanced images showed that progressive enhancement was exhibited by all patients and followed two major patterns. Four patients (4/9, 44.44%) showed ring enhancement with hypovascularity, and five patients (5/9, 55.56%) showed ring enhancement with hypervascularity. This feature has been reported in previous studies (25).

The “multilayer ring appearance” is considered a characteristic of HEHE. Previous studies have termed this ring structure as a “target-like sign” (19, 21), such as “white target-like”, and “black target-like” (26). The “white target-like sign” is defined by a nodular enhancement in the central part of the lesion in the arterial phase surrounded by ring-like enhancement in the portal venous and delayed phases (26). The “black target-like” sign correlates with peripheral enhancement with central low signal intensity in the arterial phase and enhanced lesions surrounded by a thin hypointense ring in the portal venous and delayed phases (26). However, we found that the “target-like sign” did not comprehensively reflect the characteristics of the lesions. Lesions had a circular structure with multiple layers and alternation between high and low signal intensity. Therefore, we named it the “multilayer ring appearance”, which emphasized the difference in the number of layers, the difference between each layer, and the characteristics of the ring-like structures.

We analyzed the multilayer ring structure of 121 lesions in nine patients and found that most lesions had a two-layer structure with a maximum of four layers. Several patients exhibited two patterns of the “multilayer ring appearance” (mainly differences in the number of layers). Moreover, we compared the appearance of the feature using different imaging sequences and found that the “multilayer ring appearance” could be detected more clearly in T2WI. Notably, there were significant differences in the size of lesions between different layers of the “multilayer ring appearance” (*p* < 0.001). This suggested that the number of layers in the “multilayer ring appearance” of the lesion is related to the growth of the lesion, where the larger the lesion, the higher the number of layers. Therefore, further pathological studies are needed to better understand the “multilayer ring appearance” of HEHE. However, irrespective of the number of layers of ring structure in the lesion, the DWI images always showed a two-layer target structure with a hypointense inner layer and core and a hyperintense rim.

Imaging features are often related to the molecular mechanism of the tumor. The occurrence and progression of liver tumors have complex molecular and cellular changes, which can be useful for understanding the biological behavior and the treatment of tumors (27–30). Therefore, in terms of the specific imaging characteristics of HEHE, we can empirically deduce that the tumor cells around the lesion have more active growth status and are involved in cellular proliferation, whereas tumor cells in the inner layer and center are inactive. Some studies found that most of the nodules had a growth pattern with infiltrative margins by pathological examination (7). If further pathological and molecular biological experiments can confirm this finding, there is great promise for the development of new treatment methods for HEHE.

The relationship between lesions and blood vessels may be another feature of HEHE. Several studies have suggested that the “lollipop sign” (12, 21), which starts from the portal vein and terminates around the lesion, represents the lesion (candy in the lollipop) and the adjacent vein (the stick) (12, 21). Few studies have suggested that blood vessels enter the interior part of the lesion. However, our study of 121 lesions showed no obvious blood vessel penetration (blood vessels that penetrate lesions and extend a certain distance) in five smaller lesions (less than 0.6 cm, which was considered related to the size of lesions and scanning section), whereas blood vessel penetration was observed in the remaining 116/121 lesions (95.87%). We named this feature the “vascular penetration sign”, which differs from the “lollipop sign”. The major blood vessels involved were portal veins (92.56%) and small branches of the terminal, and a few patients had the “vascular penetration sign” of the hepatic vein. No other vessels were found. This suggested that the main reason for multiple lesions is that tumor cells migrate along with the intrahepatic portal vein blood flow; however, this warrants further study. In addition, further study on the molecular mechanism of the formation of this vascular-related feature may provide a basis for the application of molecular target drugs, such as sorafenib, which is one of the most widely used targeted drugs for liver cancer (28, 31).

Interestingly, 3/9 patients had extrahepatic metastases, which were mainly found in the lungs. These three patients all had the “vascular penetration sign” of the hepatic vein. Tumor cells may enter the inferior vena cava through the hepatic vein and subsequently reach the lungs, which may cause pulmonary metastasis. Our MRI analysis of the other six patients with no pulmonary metastasis showed that all the blood vessels of lesions with the “vascular penetration sign” were portal veins rather than hepatic veins. Therefore, we speculated that the “vascular penetration sign” of the hepatic vein is related to lung metastasis; moreover, tumor cells may infiltrate the vascular walls to metastasize. An international multicenter study showed positive macrovascular invasion in surgical specimens and extrahepatic disease (7).

In conclusion, this study investigated the MRI findings of nine patients with the rare disease HEHE in combination with clinical practice and found distinctive features, which included the “multilayer ring appearance”, “vascular penetration sign”, and two patterns of enhancement. The “vascular penetration sign” of the hepatic vein was highly indicative of lung metastasis. These findings are of great significance for the diagnosis and treatment of HEHE; moreover, they provide insight into the development and prognosis of the disease. Further comparative studies of pathology during different stages of disease progression are needed to gain a better understanding of HEHE.

## Data Availability Statement

The original contributions presented in the study are included in the article/supplementary material. Further inquiries can be directed to the corresponding authors.

## Ethics Statement

The studies involving human participants were reviewed and approved by The 5th Medical Center of PLA General Hospital. The patients/participants provided their written informed consent to participate in this study.

## Author Contributions

XG performed the statistical analysis and drafted the manuscript and the study design. XG, YY, WZ, HZ, YZ, KZ, HK, LY, YC, YB, and ZZ performed the MRI of the patient studies. XG, HZ, WZ, YZ, and KZ take responsibility for the data collection and sorting.

## Conflict of Interest

The authors declare that the research was conducted in the absence of any commercial or financial relationships that could be construed as a potential conflict of interest.

## Publisher’s Note

All claims expressed in this article are solely those of the authors and do not necessarily represent those of their affiliated organizations, or those of the publisher, the editors and the reviewers. Any product that may be evaluated in this article, or claim that may be made by its manufacturer, is not guaranteed or endorsed by the publisher.
